# Changes in cellular Ca^2+^ and Na^+^ regulation during the progression towards heart failure in the guinea pig

**DOI:** 10.1113/JP277038

**Published:** 2019-03-18

**Authors:** H.‐Y. Ke, H.‐Y. Yang, A. J. Francis, T. P. Collins, H. Surendran, A. Alvarez‐Laviada, J. M. Firth, K. T. MacLeod

**Affiliations:** ^1^ Cardiovascular Surgery Tri‐Service General Hospital National Defense Medical Center Taipei Taiwan (ROC); ^2^ National Heart and Lung Institute Imperial College Hammersmith Hospital Du Cane Road London W12 0NN UK; ^3^ The Wellcome Trust Gibbs Building, 215 Euston Road London NW1 2BE UK

**Keywords:** cardiac hypertrophy, heart failure, sodium, calcium, Na^+^/K^+^ ATPase

## Abstract

**Key points:**

- During compensated hypertrophy *in vivo* fractional shortening (FS) remains constant until heart failure (HF) develops, when FS decreases from 70% to 39%.
- Compensated hypertrophy is accompanied by an increase in *I*_Na,late_ and a decrease in Na^+^,K^+^‐ATPase current. These changes persist as HF develops.
- SR Ca^2+^ content increases during compensated hypertrophy then decreases in HF. In healthy cells, increases in SR Ca^2+^ content and Ca^2+^ transients can be achieved by the same amount of inhibition of the Na^+^,K^+^‐ATPase as measured in the diseased cells.
- SERCA function remains constant during compensated hypertrophy then decreases in HF, when there is also an increase in spark frequency and spark‐mediated Ca^2+^ leak.
- We suggest an increase in *I*_Na,late_ and a decrease in Na^+^,K^+^‐ATPase current and function alters the balance of Ca^2+^ flux mediated by the Na^+^/Ca^2+^ exchange that limits early contractile impairment.

**Abstract:**

We followed changes in cardiac myocyte Ca^2+^ and Na^+^ regulation from the formation of compensated hypertrophy (CH) until signs of heart failure (HF) are apparent using a trans‐aortic pressure overload (TAC) model. In this model, *in vivo* fractional shortening (FS) remained constant despite HW:BW ratio increasing by 39% (CH) until HF developed 150 days post‐TAC when FS decreased from 70% to 39%. Using live and fixed fluorescence imaging and electrophysiological techniques, we found an increase in *I*_Na,late_ from –0.34 to –0.59 A F^−1^ and a decrease in Na^+^,K^+^‐ATPase current from 1.09 A F^−1^ to 0.54 A F^−1^ during CH. These changes persisted as HF developed (*I*_Na,late_ increased to –0.82 A F^−1^ and Na^+^,K^+^‐ATPase current decreased to 0.51 A F^−1^). Sarcoplasmic reticulum (SR) Ca^2+^ content increased during CH then decreased in HF (from 32 to 15 μm l^−1^) potentially supporting the maintenance of FS in the whole heart and Ca^2+^ transients in single myocytes during the former stage. We showed using glycoside blockade in healthy myocytes that increases in SR Ca^2+^ content and Ca^2+^ transients can be driven by the same amount of inhibition of the Na^+^,K^+^‐ATPase as measured in the diseased cells. SERCA function remains constant in CH but decreases (τ for SERCA‐mediated Ca^2+^ removal changed from 6.3 to 3.0 s^−1^) in HF. In HF there was an increase in spark frequency and spark‐mediated Ca^2+^ leak. We suggest an increase in *I*_Na,late_ and a decrease in Na^+^,K^+^‐ATPase current and function alters the balance of Ca^2+^ flux mediated by the Na^+^/Ca^2+^ exchange that limits early contractile impairment.

## Key points

- During compensated hypertrophy *in vivo* fractional shortening (FS) remains constant until heart failure (HF) develops, when FS decreases from 70% to 39%.
- Compensated hypertrophy is accompanied by an increase in *I*_Na,late_ and a decrease in Na^+^,K^+^‐ATPase current. These changes persist as HF develops.
- SR Ca^2+^ content increases during compensated hypertrophy then decreases in HF. In healthy cells, increases in SR Ca^2+^ content and Ca^2+^ transients can be achieved by the same amount of inhibition of the Na^+^,K^+^‐ATPase as measured in the diseased cells.
- SERCA function remains constant during compensated hypertrophy then decreases in HF, when there is also an increase in spark frequency and spark‐mediated Ca^2+^ leak.
- We suggest an increase in *I*_Na,late_ and a decrease in Na^+^,K^+^‐ATPase current and function alters the balance of Ca^2+^ flux mediated by the Na^+^/Ca^2+^ exchange that limits early contractile impairment.

## Introduction

In the heart the regulation of intracellular Ca^2+^ is tightly coupled to the regulation of intracellular Na^+^ because of the existence of the Na^+^/Ca^2+^ exchange located in the sarcolemmal membrane (Reuter & Seitz, 1968; Mullins, 1981). The primary ‘beat‐to‐beat’ function of the exchange is to remove an amount of Ca^2+^ from the cytoplasm equivalent to that which enters the cell during the early part of the action potential to trigger sarcoplasmic reticulum (SR) Ca^2+^ release, thereby maintaining cell Ca^2+^ homeostasis (Bridge *et al*. 1990; Bassani *et al*. 1994; Bers, 2002). The operation of the exchange is controlled in part by the intracellular Na^+^ concentration ([Na^+^]_i_), which in turn is determined by the balance between Na^+^ influx (through Na^+^ channels and transporters) and Na^+^ efflux (mediated by the Na^+^,K^+^‐ATPase also located in the surface membrane) (Bers *et al*. 2003; Despa & Bers, 2013)

An increase in [Na^+^]_i_ will alter the balance of Ca^2+^ flux generated by the Na^+^/Ca^2+^ exchange during the cardiac cycle increasing reverse mode behaviour that could supply more Ca^2+^ influx and serve as a compensation system for the reduced contractility and cardiac function that has been observed in the hypertrophic heart and is a well‐known characteristic of the failing heart. The compensation may occur at the expense of increasing the likelihood of arrhythmogenesis (Pogwizd *et al*. 1999, 2001) and activation of hypertrophic and apoptotic signalling (Molkentin, 2004).

Increased [Na^+^]_i_ is a general finding associated with cardiac hypertrophy (Jelicks & Siri, 1995; Gray *et al*. 2001) and heart failure (Despa *et al*. 2002; Pieske *et al*. 2002; Baartscheer *et al*. 2003; Schillinger *et al*. 2006; Liu *et al*. 2014) but there have been very few studies that have measured the function of the Na^+^,K^+^‐ATPase in these pathological conditions and such studies vary in their results and conclusions perhaps depending on the animal model used.

For example, ventricular myocytes isolated from rabbit hearts in which failure was induced by aortic insufficiency and constriction showed no difference in *V*_max_ or *K*_m_ of Na^+^,K^+^‐ATPase‐mediated Na^+^ extrusion compared with control cells (Despa *et al*. 2002). The authors concluded that [Na^+^]_i_ is increased in heart failure (HF) as a result of higher Na^+^ influx possibly through a tetrodotoxin‐sensitive pathway or up‐regulation of the Na^+^/H^+^ exchange (Baartscheer *et al*. 2003). In contrast, another study found that ventricular myocytes isolated from failing hearts induced by myocardial infarction (ligation of the left coronary artery) were slower to extrude Na^+^ compared with those from sham hearts, but there was no change in affinity of the ATPase for Na^+^ despite evidence for a switch in isoform expression (Semb *et al*. 1998). Verdonck *et al*. (2003 *a*) found no evidence for isoform switching, no change in maximum pump current but a decrease in the sensitivity of Na^+^,K^+^‐ATPase for Na^+^ in myocytes isolated from hypertrophied canine hearts subjected to complete atrioventricular block.

Most studies that have investigated the changes in cellular Ca^2+^ and Na^+^ regulation in the hypertrophic or failing heart have done so by comparing ventricular myocytes isolated from control hearts with those isolated from hearts at one time point, either in a hypertrophic or in a failing state. In this study we follow the changes in cellular Ca^2+^ and Na^+^ regulation from the initial stages of cardiac overload and the formation of compensated hypertrophy, until signs of heart failure are apparent in an animal whose cardiac cell physiology closely resembles that of the human. We consider a variety of indices that dictate heart function and intracellular Ca^2+^ and Na^+^ regulation to build a picture of the progression of cellular Ca^2+^ pathology. Our results suggest a continuum of change with one of the earliest modifications being a reduction in Na^+^,K^+^‐ATPase current.

## Methods

### Ethical approval

Male Dunkin–Hartley guinea pigs purchased from Marshall BioResources (Hull, UK) were used and housed at 21 ± 1°C on a 12h light–dark cycle and provided with standard guinea pig feed and water *ad libitum*. All studies were carried out with the approval of the Animal Welfare and Ethical Committee of Imperial College and the Home Office, UK and are in accordance with the UK Home Office *Guidance on the Operation of the Animals (Scientific Procedures) Act 1986*, which conforms to the *Guide for the Care and Use of Laboratory Animals* published by the US National Institutes of Health under assurance number A5634‐01.

### Animal model

Cardiac hypertrophy was induced using the surgical procedure described by Kingsbury *et al*. (2000) but performed on animals weighing 350–450 g and with some modifications to the Teflon constrictor and anaesthetic regime. Anaesthesia was induced with 4% isoflurane in 100% oxygen (Isoflo, Abbott, Maidenhead, UK). Once animals were anaesthetized, they were given atropine sulfate 0.05 mg kg^−1^ s.c. (Atrocare, Animalcare Ltd, York, UK) to reduce the oral and nasal secretions and facilitate endotracheal intubation. Lidocaine (Xylocaine 10 mg, AstraZeneca, Cambridge, UK) spray was applied prior to endotracheal intubation which was with a 14G × 64 mm intravenous catheter. After intubation, anaesthesia was maintained with 1.5∼2% isoflurane in oxygen via the endotracheal tube. Animals were ventilated with a Zoovent Mini ventilator (Triumph Technical Services, Milton Keynes, UK) at 70 ml kg^−1^ and 70 cycles min^−1^. End tidal CO_2_, peripheral O_2_ saturation and pulse rate were monitored with a Capnovet‐10 (Vetronic Services Ltd, Newton Abbot, UK) and pulse‐oxymeter (SurgiVet, Smiths Medical, Dublin, OH, USA). Prophylactic antibiotic (enrofloxacin (Baytril, Bayer, Leverkusen, Germany) 5 mg kg^−1^ s.c.) and analgesia (carprofen (Rimadyl, Pfizer, Surrey, UK) 5 mg kg^−1^ s.c. and buprenorphine 0.05 mg kg^−1^ s.c.) were given after stable anaesthesia had been achieved. Local anaesthetic was administered (bupivacaine 2 mg kg^−1^; Vetergesic, Alstoe Animal Health, York, UK) by subcutaneous injection around the incision site and intraoperative hydration was provided with 4 mg kg^−1^ h^−1^ saline 0.9% s.c. carprofen (Rimadyl, 0.05 mg ml^−1^) was given orally for 3 days to maintain post‐operative analgesia.

M‐mode echocardiographic measurements were carried out on un‐anaesthetized animals to overcome anaesthetic drug influences on heart function. An S‐12 paediatric echo probe on an Agilent HP 5500 (Agilent Technologies, Santa Clara, CA, USA) cardiac echo machine was used to assess the degree of ventricular function and left ventricular hypertrophy *in vivo*.

### Single cell experiments

Experiments were carried out on isolated left ventricular myocytes enzymatically dissociated from sham‐operated and aortic‐banded guinea pig hearts as already described (MacLeod & Harding, 1991) but using the Liberase TL enzyme blend (Roche Diagnostics GmbH, Mannheim, Germany). For the single cell isolations, the heart and lungs were rapidly explanted from the thorax of anaesthetized animals (4% isoflurane in 100% oxygen) and stored in ice‐cold Krebs–Henseleit solution (composition below) containing 500 IU heparin sodium.

The Ca^2+^‐sensitive fluorescent dye fluo‐4 was used to monitor changes in intracellular [Ca^2+^]. To load the myocytes with the dye, 1 ml aliquots of cell suspension were incubated with 10 μm of the acetoxymethyl ester form (Invitrogen, Life Technologies Ltd, Paisley, UK) for 25 min at room temperature (RT). Once loaded, cells were not used for at least another 30 min to allow the intracellular fluo‐4‐AM to be de‐esterified.

Left ventricular myocytes were stored in Dulbecco's modified Eagle's medium (Gibco BRL, Life Technologies Ltd) at RT until used. During storage, the cells were protected from light to avoid bleaching of the fluorescent indicator. A drop of cell suspension was placed onto a glass coverslip that formed the floor of a fast exchange, open diamond RC24N superfusion chamber (Warner Instruments, Hamden, CT, USA). The chamber was mounted on the stage of an inverted Nikon Eclipse or Diaphot microscope and the cells visualized with a Nikon ×40 fluor oil immersion lens (NA = 1.3). Cells were superfused with normal Tyrode solution (composition below) at 37°C. Cells with rounded edges, obvious cytoplasmic vesicles, automatic activity prior to stimulation, major ultrastructural defects, absence of clear striations or cells that were not incompletely isolated (e.g. cell pairs) were not used.

Ca^2+^ sparks were recorded by previously described methods and analysis of the line scan images was performed using ImageJ (NIH, Bethesda, MD, USA, RRID:SCR_003070) with the SparkMaster plugin (Picht *et al*. 2007) and custom macros (Sikkel *et al*. 2013). Detection criteria for Ca^2+^ sparks were set at 4.2 times the standard deviation above the mean background value. Spark mass was calculated using the formulae derived by Hollingworth *et al*. (2001). To assess spark characteristics, myocytes were initially paced at 0.5 Hz before increasing the frequency to 2 Hz for 20 s. Field stimulation was then stopped, and sparks recorded during a quiescent 30 s period.

Action potential duration and Na^+^/Ca^2+^ exchange currents and steady‐state changes to Na^+^,K^+^‐ATPase currents were recorded using switch‐clamping (AxoClamp 2B amplifier, Molecular Devices, Sunnyvale, CA, USA) with high resistance microelectrodes to minimize cell dialysis so preserving the intracellular milieu. Sharp microelectrodes had resistances of 20–30 MΩ and were filled with a solution that contained 2 m KCl, 10 mm Hepes, 100 μm EGTA, pH adjusted to 7.20 with KOH. The required signals were recorded using pClamp (v 8 or 10) acquisition and analysis software (Molecular Devices, RRID:SCR_011323).

Reactivation of Na^+^,K^+^‐ATPase currents following pump block and late sodium currents were measured by using the whole‐cell patch‐clamp technique to improve cytoplasmic ionic control. The resistances of the patch‐pipettes ranged from 4 to 7 MΩ. Pipettes for sharp electrodes and patch experiments were pulled from glass capillaries 1.5 mm OD and 0.86 mm ID with an internal glass filament.

### Solutions

Normal Tyrode (NT) contained, in mm: NaCl 140, KCl 6, CaCl_2_ 2, MgCl_2_ 1, glucose 10, Hepes 10; pH adjusted to 7.4 with NaOH. Krebs–Henseleit buffer contained, in mm: NaCl 119, KCl 4.7, MgSO_4_ 0.94, CaCl_2_ 1, KH_2_PO_4_ 1.2, NaHCO_3_ 25, glucose 11.5; pH adjusted to 7.4 with HCl. Tris‐buffered saline and Tween 20 (TBST) for 1 l contained, 100 ml of TBS 10× (Bio‐Rad, Watford, UK, no. 1706435), 900 ml of distilled water and 1 ml Tween 20. The external solution for Na^+^,K^+^‐ATPase reactivation experiments contained, in mm: NaCl 140, KCl 6 (or 0 for inhibition of the Na^+^,K^+^‐ATPase), glucose 10, Hepes 10, MgCl_2_ 1, BaCl_2_ 2, NiCl_2_ 5; pH adjusted to 7.4 with NaOH. The pipette filling solution for Na^+^,K^+^‐ATPase reactivation experiments contained, in mm: caesium methane sulfonate 100, NaCl 30, CsCl 10, Hepes 10, EGTA 5, Mg‐ATP 5, MgCl_2_ 0.75; pH adjusted to 7.2 with CsOH. The superfusing (external) solution for late sodium current experiments contained, in mm: NaCl 137, CsCl 5.4, glucose 10, Hepes 10, MgCl_2_ 2, CaCl_2_ 1.8, nitrendipine 0.002; pH adjusted to 7.4 with CsOH. The pipette filling solution for late sodium current experiments contained, in mm: caesium methane sulfonate 100, NaCl 10, CsCl 30, Hepes 10, EGTA 5, Mg‐ATP 5, MgCl_2_ 0.75; pH adjusted to 7.2 with CsOH.

When required, cells were externally field‐stimulated by a 5 ms bipolar pulse at 1.5 times threshold voltage delivered through platinum electrodes placed on each side of the superfusion chamber. Action potential duration, Ca^2+^ transients and quantitative determinations of SR Ca^2+^ content were made using well described techniques currently in use in the laboratory (MacLeod & Harding, 1991; Terracciano *et al*. 1997, 1998; Sikkel *et al*. 2013).

### Electrophysiology protocols

To assess the relaxation contribution from Na^+^/Ca^2+^ exchange, SERCA and other slow components (thought to include the sarcolemma Ca^2+^‐ATPase and mitochondria Ca^2+^ uniport), a standard single exponential equation was used to fit the decay trace of a steady‐state Ca^2+^ transient to obtain a time constant (τ) that represented the combined contributions of the Ca^2+^ regulatory mechanisms. Cells were then exposed to NT containing 10 mm caffeine (at a high flow rate) to induce a release of Ca^2+^ from the SR. The decay phase of this caffeine‐induced transient represented the contribution of Na^+^/Ca^2+^ exchange and slow components. Removal of the caffeine followed by external field stimulation at 0.5 Hz ensured the cell returned to steady‐state. Caffeine was rapidly applied a second time but in Na^+^‐free, Ca^2+^‐free solution to elicit another caffeine‐induced Ca^2+^ transient. In this case, Na^+^/Ca^2+^ exchange was blocked by the Na^+^‐free, Ca^2+^‐free solution, and therefore the decay τ of the second caffeine‐induced transient represented the contribution of the slow components to Ca^2+^ efflux. The rate constants of each decay phase were calculated as 1/τ.

Na^+^,K^+^‐ATPase current was measured in cells voltage‐clamped at –80 mV. First a train of preconditioning pulses (square clamp pulses from –80 to 0 mV for 300 ms at a frequency of 1 Hz) were applied for 1 min to ensure steady‐state [Na^+^]_i_ had been reached. Stimulation was stopped and the superfusate switched to NT containing 2 mm BaCl_2_ and 5 mm NiCl_2_ (Ba^2+^ was used to block K^+^ channels and Ni^2+^ to block Ca^2+^ channels and inhibit the Na^+^/Ca^2+^ exchange). After 1 min, 5 μm dihydro‐ouabain (DHO) was applied to inhibit the high affinity component of the Na^+^,K^+^‐ATPase and after a further 1 min, 500 μm strophanthidin was applied to produce complete inhibition of the current so allowing measurement of total pump current and that due to the individual isoforms of the Na^+^,K^+^‐ATPase.

Late Na^+^ current (*I*_Na,late_) was recorded at RT using a modified protocol established from previous work (Maltsev *et al*. 1998; Toischer *et al*. 2013). Pipette and external solutions were caesium‐based to block K^+^ currents. Cells were subjected to a depolarizing step to –20 mV for 2 s from a holding potential of –120 mV (at a rate of 2 Hz) to induce Na^+^ current. This sequence was repeated following superfusion of NT containing 10 μm ranolazine for 3 min. *I*_Na,late_ was obtained by subtracting the trace in the presence of ranolazine from the original trace. *I*_Na,late_ density was determined from the averaged current measured between 210 and 220 ms after the depolarizing step to –20 mV. This time interval was chosen to avoid a contribution of the transient (early) Na^+^ current which is reported to be completely inactivated within 200 ms (Aistrup *et al*. 2013).

### Immunocytochemistry experiments

Enzymatically isolated left ventricular myocytes were allowed to attach (1 h, 37°C) to glass bottom culture dishes (MatTek Corporation, MA, USA) pre‐coated with laminin (Gibco, Thermo Fisher Scientific, Rochford, UK; no. 23017015). They were washed with phosphate buffered saline (PBS) three times then fixed with 4% paraformaldehyde (PFA) for 10 min and permeabilized with Triton X‐100 (0.05% in PBS for 10 min). Non‐specific antibody binding sites were blocked by incubating the cells in PBS containing 5% bovine serum albumin (BSA) and 3% goat serum for 30 min before application of the primary antibodies. These procedures were carried out at RT.

Primary antibodies (mouse anti‐Na^+^,K^+^‐ATPase α1, Invitrogen, cat. no. MA1‐16731, lot no. RK2298051 and rabbit anti‐Na^+^,K^+^‐ATPase α2, Merck, Kenilworth, NJ, USA; cat. no. AB9094‐I, lot no. 2855648) were added at dilutions (with blocking solution with PBS) of 1:200 and 1:150, respectively, and the cells incubated overnight at 4°C. The myocytes were then washed 3 times with PBS for 5 min. Alexa Fluor 488 conjugated secondary antibodies (Alexa Fluor 488 goat anti‐rabbit IgG, Invitrogen cat. no. A11008, lot no. 1124089, and Alexa Fluor 488 goat‐anti mouse IgG, Invitrogen cat. no. A11029, lot no. 1073083) were used accordingly at 1:500 dilution and applied to the cells for 3 h. After washing the cells, glycerol‐based mounting medium containing 4′,6‐diamidino‐2‐phenylindole (DAPI) (Vector Laboratories Inc., Burlingame, CA, USA; cat. no. H‐1000, lot no. ZC1216) was added. Image acquisition was performed with a Zeiss LSM780 confocal microscope (Zeiss, Oberkochen, Germany). The settings on the confocal microscope were standardized at 1 Airy unit and gain and offsets were kept constant for all image collections and measurements for each antibody. Cell images were recorded approximately mid‐way through the cell depth as judged by DAPI staining. Two regions of interest (ROI) (143 × 93 pixels) were analysed using ImageJ; one was placed over the sarcolemmal area and the other in the centre of each cell. The image threshold was set to allow calculation of the area of fluorescence of the antibody, minimizing the acquisition of background staining. The mean area of fluorescence was calculated from the two ROIs for each cell and the measurements pooled with results from at least 10 cells from two hearts of each group.

### Western blotting

SDS‐PAGE and western blotting experiments were carried out using standard techniques using the Bio‐Rad mini gel system. Different percentage gels were used for specific proteins of interest: 8% gels for Na^+^,K^+^‐ATPase α1 and α2 and 12% gels for phospholemman.

The membrane containing the diluted proteins was blocked with blocking grade buffer (Bio‐Rad cat. no. 1706404) overnight at 4°C to prevent non‐specific binding of the detection antibodies during the subsequent steps. The membrane was incubated with either Na^+^,K^+^‐ATPase α1 primary antibody (Invitrogen cat. no. MA1‐16731, lot no. RK2298051) at 1:2000 dilution or glyceraldehyde 3‐phosphate dehydrogenase (GAPDH) primary antibody (Santa Cruz Biotechnology, Dallas, TX, USA, cat. no. sc‐47724, lot no. F2316) at 1:10000 dilution for 1 h at RT. Following a series of washing steps with TBST, the membrane was incubated with secondary antibody (anti‐mouse IgG–horseradish peroxidase (HRP), Abcam, Cambridge, UK, cat. no. ab205719, lot no. GR247077‐7) at 1:2000 dilution for 1 h at RT. The membrane was subsequently washed again and the HRP‐conjugated antibody bound to the target protein was visualized by Clarity Western ECL Substrate (Bio‐Rad, cat. no. 1705061) and captured by a G Box Chemi XT 16 (SynGene, Cambridge, UK) (4 min exposure) using GeneSnap software version 7.12.06 (SynGene, RRID:SCR_014249).

For the Na^+^,K^+^‐ATPase α2 western blot, the membrane was blocked for 1 h at RT and then incubated with the α2 antibody (Merck, cat. no. AB9094‐I, lot no. 2855648) overnight at 4°C. Following a series of TBST washing steps, the NKAα2 membrane was incubated with anti‐rabbit IgG‐HRP secondary antibody (Cell Signaling Technology, Danvers, MA, USA, cat. no. 7074S, lot no. 26) and the GAPDH was incubated with anti‐mouse IgG‐HRP, both at 1:2000 dilution for 1 h at RT. The membrane was washed again and developed using Clarity Western ECL with exposure time of 5 min. A similar protocol was used for the phospholemman (PLM) blot. The membrane was blocked for 1 h at RT and incubated in primary antibody anti‐rabbit FXYD1 (Abcam, cat. no. ab76597, lot no. GR260084‐1) overnight at 4°C. The membrane was washed using TBST and incubated with secondary antibody (anti‐rabbit IgG‐HRP) at 1:2000 dilution for 1 h at RT. The membrane was washed and developed using Clarity Western ECL with exposure time of 2 min.

Western blotting was carried out using a minimum of *n* = 4 samples for each group investigated. Each blot was repeated three times and the averaged density value for each sample was used to determine differences between means. Representative samples are illustrated in the final western blot. ImageJ software was used to analyse the blots.

### Statistical tests

Data are presented as mean (SD) or mean (95% confidence intervals) and *P* values are designated with asterisks: ^***^ *P* < 0.001, ^**^ *P* < 0.01, ^*^ *P* < 0.05. Either Student's *t* test or ANOVA was used to calculate *P* values with Prism 7 software (GraphPad Software Inc., San Diego, CA, USA). Experimental values were normally distributed except some Ca^2+^ spark data that were transformed logarithmically where necessary before running the statistical test. The number of cells used in each study together with the number of hearts from which they were isolated are detailed in the figure legends.

Experimenters were blinded to the origin of the cells used in the immunocytochemistry experiments but in all other studies experimenters were not blinded.

## Results

### Whole heart function

This animal model of pressure overload due to transverse aortic constriction (TAC) has been carefully studied at the point where cardiac hypertrophy is well developed – between 40 and 60 days post‐operatively (Cooklin *et al*. 1997; Kingsbury *et al*. 2000; Gray *et al*. 2001). Our aim with the work detailed here was to investigate how intracellular Ca^2+^ and Na^+^ regulation changes as the hearts progress from established hypertrophy to HF.

**Figure 1:**
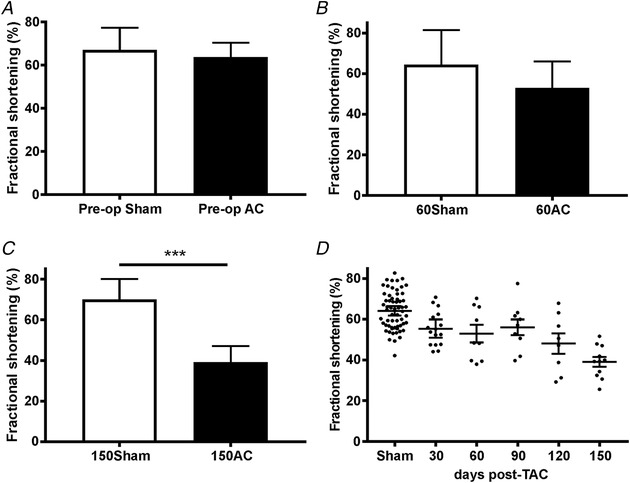
**The changes to fractional shortening (FS) just prior to the AC surgery (pre‐operation) (*A*), at 60 days post‐AC (*B*) and at 150 days post‐AC (*C*)** Number of animals (*N*) for Pre‐op = 19 sham, 39 AC; *N* for 60 day = 7 sham, 9 AC; and *N* for 150 day = 9 sham, 11 AC. *A*–*C* show mean (SD). *C*: ^***^ *P* < 0.001, Student's *t* test. *D*, the decline in FS over time in the form of scatter plots in which the longer bars show mean and the shorter bars 95% CI. In this panel the sham values have been combined because there are no significant differences in FS in sham animals over time.

The first task was to assess *in vivo* cardiac function over a longer period of time post‐operatively. Figure 1 shows the changes to fractional shortening from just prior to the aortic constriction (AC) surgery to 150 days post‐AC. Fractional shortening (FS) is an index of the size reduction of the left ventricle calculated by the following equation: FS = (LVIDd − LVIDs/LVIDd) × 100, where LVIDd is left ventricular internal dimension during diastole and LVIDs is the same dimension during systole. FS reflects left ventricular systolic function. These data suggest this index is not significantly different between sham and AC animals until 150 days post‐AC.

LVIDs and LVIDd provide an assessment of chamber dilatation, which is a well‐recognized precursor of ventricular dysfunction and heart failure. These indices are also not significantly different between sham and AC animals until 150 days post‐AC (Table 1). The heart‐to‐body weight ratio (HW:BW) is the most commonly used index of cardiac hypertrophy. A significant increase in HW:BW occurs at 60 and 150 days post‐AC, suggesting cardiac hypertrophy develops around 60 days and is maintained for at least 90 days thereafter.

**Table 1:** Left ventricular internal diameter during systole (LVIDs) and during diastole (LVIDd), heart to body weight ratios (HW:BW) and lung to body weight ratios (LW:BW) just before the AC operation and days thereafter

Days post‐AC		0	30	60	90	120	150
LVIDs (cm)	Sham	0.19 (0.06) [10]	0.22 (0.05) [7]	0.28 (0.16) [5]	0.28 (0.07) [6]	0.26 (0.05) [6]	0.18 (0.02) [5]
	AC	0.19 (0.07) [56]	0.30 (0.08) [17]	0.29 (0.08) [8]	0.29 (0.09) [9]	0.35 (0.16) [10]	0.50 (0.10) [11]***
LVIDd (cm)	Sham	0.55 (0.13) [10]	0.61 (0.05) [7]	0.60 (0.11) [5]	0.67 (0.05) [6]	0.63 (0.07) [6]	0.66 (0.10) [11]
	AC	0.53 (0.07) [56]	0.65 (0.08) [17]	0.62 (0.11) [8]	0.66 (0.06) [9]	0.72 (0.09) [10]	0.79 (0.10) [11]**
HW:BW (g kg^−1^)	Sham		3.97 (0.24) [7]	3.51 (0.18) [5]			3.45 (0.25) [5]
	AC		5.14 (2.23) [17]	5.20 (0.57) [8]***			4.80 (0.96) [11]***
LW:BW (g kg^−1^)	Sham		5.93 (0.54) [9]	4.88 (0.46) [15]			4.58 (0.67) [20]
	AC		7.08 (3.11) [6]	7.08 (3.42) [11]			6.90 (3.20) [16]*

Values are expressed as means (SD) and [*N*] = number of animals. ^*^ *P* < 0.05 150 day LW:BW sham *vs*. AC. ^**^ *P* < 0.01 LVIDd sham *vs*. AC. ^***^ *P* < 0.001 LVIDs sham *vs*. AC and *P* < 0.001 60 day and 150 day HW:BW sham *vs*. AC.

The mean lung‐to‐body weight ratio (LW:BW) also increases 150 days post‐AC. No changes to LW:BW occur at earlier time points. The lung weight changes correlate well with the increase in LVIDd, which is also only different at 150 days post‐AC. The enlarged LVIDd indicates volume retention in the left ventricle and this is likely to produce pulmonary congestion confirmed by the increased LW:BW at this stage.

We conclude from these whole heart measurements that between 30 and 60 days post‐AC there is no decrease in heart function, yet heart weight has increased suggesting that compensated hypertrophy has occurred. At 150 days post‐AC the compensation has halted, whole heart function is poor and signs of heart failure are apparent. To assess changes to Ca^2+^ and Na^+^ regulation in cardiac myocytes we isolated single cells at two time points – at 60 days post‐AC (60AC), corresponding to a compensated hypertrophy stage, and at 150 days post‐AC (150AC), a time when the hearts are beginning to fail.

### Ca^2+^ transients and the SR

**Figure 2:**
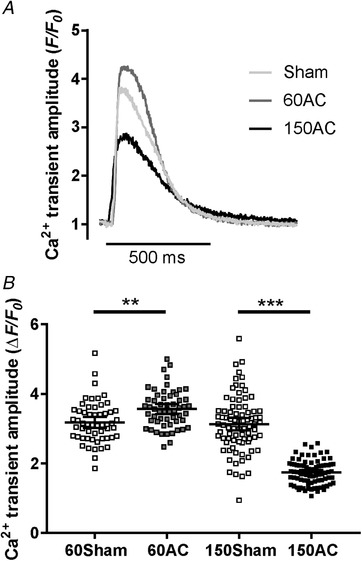
**Comparisons of amplitudes of Ca^2+^ transients** *A*, sample Ca^2+^ transients recorded from ventricular myocytes isolated from hearts 60 and 150 days after AC (60AC and 150AC, respectively) and 60 days following a sham operation (Sham). *B*, pooled data with 95% CI. Ca^2+^ transient amplitudes were 11% greater in 60AC compared with 60Sham but significantly reduced in 150AC compared with 150Sham (60Sham, *n*/*N* = 55/5; 150Sham, *n*/*N* = 81/6; 60AC, *n*/*N* = 56/5; 150AC, *n*/*N* = 84/6; one‐way ANOVA with Sidak *post hoc* test, ^**^ *P* < 0.01, ^***^ *P* < 0.001). n/*N*, total number of cells (*n*) from the total number of hearts (*N*).

During the compensated hypertrophy stage (60 days) the Ca^2+^ transients recorded from myocytes isolated from 60‐day AC hearts were larger than those isolated from sham‐operated control cells (Fig. 2 *B*). With progression of disease, the Ca^2+^ transients decreased by 40%. The time‐to‐peak of the Ca^2+^ transients was not different in cells from sham and AC hearts until 150 days when the cells isolated from AC hearts displayed a pronounced slowing of this parameter (from 58 (6.3) ms, to 68 (6.3) ms, *P* < 0.01, Student's *t* test).

**Figure 3:**
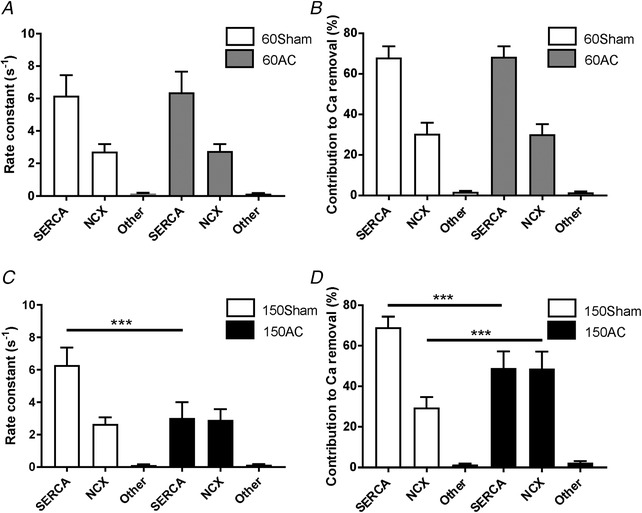
**Comparing the rates and relative contributions of the cytosolic Ca^2+^ removal mechanisms between experimental groups** *A*, the mean rate constants (SD) for 60 day hearts. 60Sham and 60AC had similar rate constants for SERCA, Na^+^/Ca^2+^ exchange (NCX) and other slower transporters. The corresponding contributions of the processes to relaxation are shown in *B*. *C* and *D*, similar measurements from 150 day hearts which show that the rate constant of SERCA is slower compared with sham. (60Sham, *n*/*N* = 55/5; 150Sham, *n*/*N* = 54/4; 60AC, *n*/*N* = 46/5; 150AC, *n*/*N* = 75/6; one‐way ANOVA with a Sidak *post hoc* test, ^***^ *P* < 0.001.)

The decline of the Ca^2+^ transients, indicating the kinetics of cytoplasmic Ca^2+^ removal, was 33% slower in 150AC compared with Sham; however, the rates were similar between Sham and 60AC groups. The rate constant of 150AC was 34% slower compared with 60AC. The rate constants of SERCA, Na^+^/Ca^2+^ exchange and other slower transporters remained unchanged between Sham and 60AC (Fig. 3 *A*) as did the contribution of each system to relaxation (Fig. 3 *B*).

By 150 days AC showed a significant increase in the rate constant of Na^+^/Ca^2+^ exchange and a 51% decrease in the rate constant of SERCA compared with those in Sham (Fig. 3 *C*). 150AC had a 28% decrease in the SERCA contribution to relaxation and 63% increase in the contribution from Na^+^/Ca^2+^ exchange (Fig. 3 *D*).

**Figure 4:**
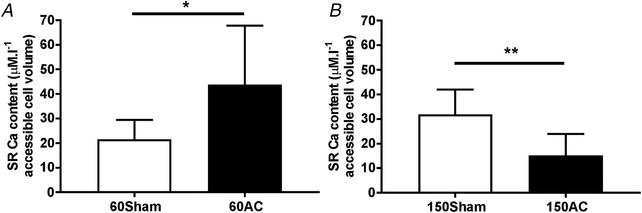
**SR Ca^2+^ content measured in left ventricular myocytes isolated from hearts 60 (*A*) and 150 days (*B*) after AC** Values plotted are mean (SD). *n*/*N* for 60 days = 17/4 (sham), 5/3 (AC); *n*/*N* for 150 days = 7/3 (sham), 8/4 (AC). Student's *t* test ^*^ *P* < 0.05, ^**^ *P* < 0.01.

SR Ca^2+^ content measured 60 days post‐AC was greater in myocytes isolated from AC hearts compared with those isolated from sham‐operated control cells (Fig. 4 *A*). With progression of disease the SR Ca^2+^ content decreased by 48% (Fig. 4 *B*). These changes mirror the alterations to the Ca^2+^ transients and suggest that the Ca^2+^ transient amplitude at the compensated hypertrophy stage is maintained at least partly by a more Ca^2+^‐loaded SR.

**Figure 5:**
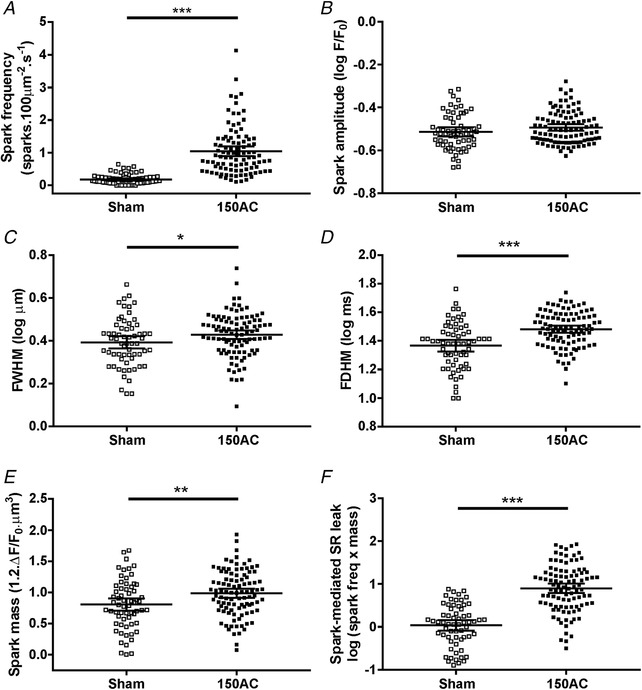
**Features of spontaneous Ca^2+^ sparks and spark‐mediated SR leak in cells isolated from the hearts of sham and 150‐day AC animals illustrated by scatter plots** Means and 95% CI are shown. *A*, Ca^2+^ spark frequency in 150‐day AC cells was significantly higher compared with 150‐day sham cells. *B*, Ca^2+^ spark amplitudes were unchanged. *C* and *D*, spark width (full width at half maximum, FWHM) was greater in 150‐day AC cells and duration (full duration at half maximum, FDHM) longer in 150‐day AC cells compared with 150‐day sham cells. *E*, 150‐day AC cells had significantly larger spark mass than 150‐day sham cells. *F*, spark‐mediated SR leak was greater in 150‐day AC cells compared with 150‐day sham cells (*n* cells/*N* hearts – 150Sham, 63/8; 150AC, 98/7; Student's *t* test, ^*^ *P* < 0.05, ^**^ *P* < 0.01, ^***^ *P* < 0.001).

At 150 days after AC we also observe changes in some of the properties of Ca^2+^ sparks (Fig. 5). Ca^2+^ spark frequency was significantly higher in cells isolated from 150‐day AC hearts compared with age‐matched sham cells. Spark mass, the volume integral of the change in fluo‐4 fluorescence, was also greater in cells isolated from 150‐day AC hearts. These data were skewed so log‐transformations were used to produce approximately Gaussian distributions. There was an increase in Ca^2+^ spark‐mediated SR leak in 150‐day AC hearts. This index is obtained by multiplying spark mass by spark frequency.

### Na^+^,K^+^‐ATPase current

**Figure 6:**
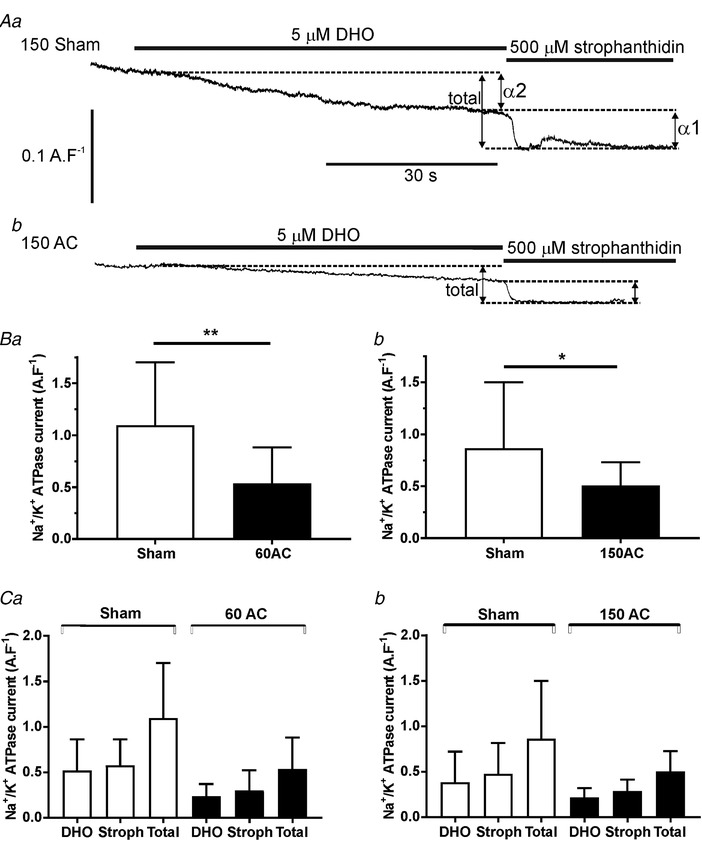
**Measurements of Na^+^, K^+^ ATPase currents and their isoforms by pharmacological inhibition** *A*, typical recordings from cells isolated from 150‐day sham (upper trace) and 150‐day post‐AC (lower trace) hearts. Dihydro‐ouabain (DHO, 5 μm) was applied to inhibit the high affinity component of the Na^+^/K^+^ (α2 isoform) and 500 μm strophanthidin was applied to produce complete inhibition of the ATPase current by inhibiting both isoforms. The individual current changes are added to produce the total current. Comparisons of the changes in total pump current between sham and AC cells are shown in *B* (*a*, 60 days post‐AC; *b*, 150 days post‐AC). Changes in α1‐ (stroph) and α2‐ (DHO) dependent currents after 60 and 150 days post‐AC are shown in *Ca* and *b*, respectively. Student's *t* test, ^**^ *P* < 0.01, ^*^ *P* < 0.05; means (SD); *n* cells/*N* hearts for 60 days = 15/6 (sham), 13/3 (AC); *n*/*N* for 150 days = 25/6 (sham), 15/4 (AC).

One possible mechanism that would explain the positive inotropy, increased Ca^2+^ transients and SR Ca^2+^ content is an inhibition of the Na^+^,K^+^‐ATPase that leads to an increase in intracellular [Na^+^]. The resultant modulation of Na^+^/Ca^2+^ exchange during the cardiac cycle would result in less Ca^2+^ being extruded from the cell and more Ca^2+^ being accumulated by the SR. Figure 6 *A* shows the strategy used to assess total Na^+^,K^+^‐ATPase current and the contribution of each isoform to the total current. The recordings are from myocytes isolated from 150‐day AC hearts (lower panel) and from 150‐day sham‐operated control hearts (upper panel). Dihydro‐ouabain (DHO, 5 μm) was applied to inhibit the high affinity component of the Na^+^,K^+^‐ATPase (α2 isoform) and 500 μm strophanthidin was applied to produce complete inhibition of the pump current. Total current declines at 60 days post‐AC (Fig. 6 *Ba*) corresponding with the increase in SR Ca^2+^ content and the compensated hypertrophy stage. The Na^+^,K^+^‐ATPase current remains low during the progression to heart failure (Fig. 6 *Bb*). We did not find any change in the ratio of isoform contribution to total current at any time during the progression to heart failure (Fig. 6 *Ca* and *b*).

**Figure 7:**
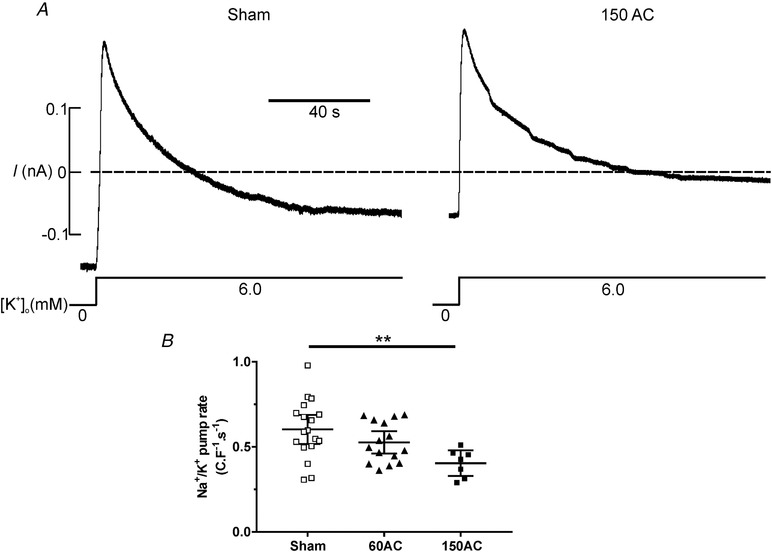
**The function of the Na^+^, K^+^ ATPase, following pump reactivation, between experimental groups** *A*, when the Na^+^,K^+^‐ATPase was inhibited by superfusion of the cells in K^+^‐free solution, the [Na^+^]_i_ is expected to increase. After 2 min the superfusate was switched to NT containing 6 mm K^+^ to reactivate the Na^+^,K^+^‐ATPase. Reactivation extrudes the accumulated Na^+^ from the cell and this rate of extrusion was used to assess the function of the Na^+^,K^+^‐ATPase. *B*, pump current measured in myocytes isolated from hearts at 150 days post‐AC (150AC) had reduced Na^+^ extrusion rates compared with the sham group. There was no change in Na^+^ extrusion rates in the age‐matched sham myocytes so these data were combined. One‐way ANOVA with a Sidak *post hoc* test ^**^ *P* < 0.01; *n* = 18, 15 and 7 for sham, 60AC and 150AC, respectively from 4–8 hearts. Scatter plots show mean (95% CI).

The Na^+^/K^+^ pump activity can be described in terms of the rate of decline of Na^+^,K^+^‐ATPase current following reactivation after a period of pump inhibition. We inhibited the pump in myocytes isolated from sham and 150‐day AC hearts with K^+^‐free superfusate (Fig. 7 *A*). The pump was then reactivated by adding back 6 mm K^+^ and this produced an outward current that decayed to a steady state. The decay phase represents the decrease in intracellular [Na^+^] that occurs on pump reactivation and therefore indicates net Na^+^ extrusion. The amount of Na^+^ pumped from the cells was therefore calculated as the integral of the current to the point where it had reached a steady state (Despa & Bers, 2003). The steady state phase is assumed to start when equilibrium is reached, i.e. when passive Na^+^ influx is balanced by the pump current. The pump rate in failing myocytes was about 65% of that in sham myocytes (Fig. 7 *B*).

The foregoing experiments present evidence of a decline in Na^+^,K^+^‐ATPase current and Na^+^ extrusion during the progression towards heart failure. To assess if the decline in these two indices of function may be explained by a decrease in the amount of pump protein, we undertook immunocytochemistry and western blotting experiments.

### Immunocytochemistry and protein expression

**Figure 8:**
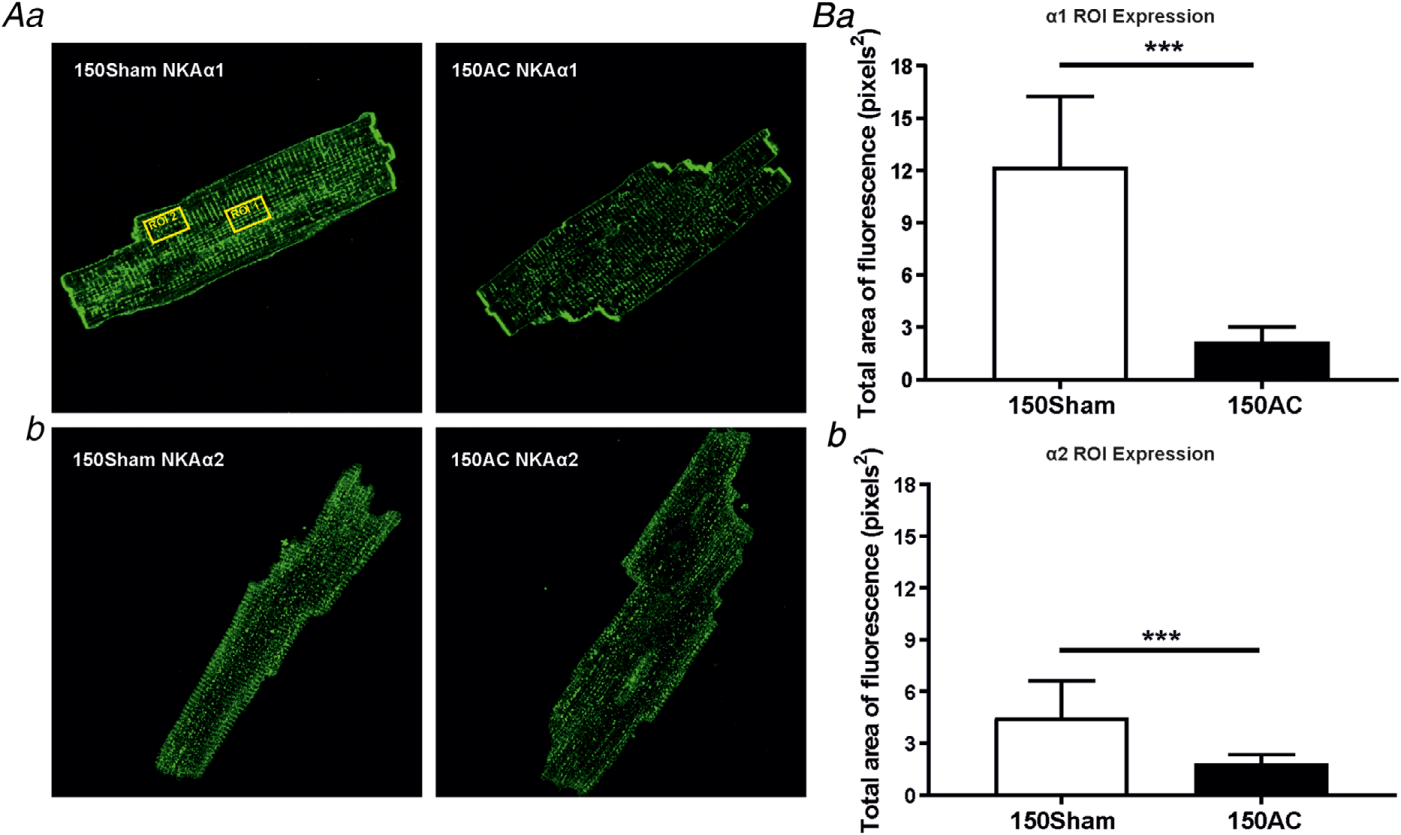
**Immunostaining** **of Na^+^, K^+^‐ATPase (NKA) isoforms α1 (*Aa* and *Ba*) and α2 (*Ab* and *Bb*)** *Aa*, cardiomyocyte staining of Na^+^,K^+^‐ATPase α1 in Sham and 150‐day post‐AC cells. Visibly reduced staining in AC cell compared to Sham. One ROI (shown by yellow rectangle 24 × 15 μm) was placed in the centre and another near the edge of cell to measure the total area of fluorescence and the mean total area of the two ROI was calculated. *b*, Na^+^,K^+^‐ATPase α2 immunolabelling in Sham and AC cells. *Ba*, total area of fluorescence of Na^+^,K^+^‐ATPase α1 markedly declines between Sham and AC. *b*, the total area of fluorescence of Na^+^,K^+^‐ATPase α2 is significantly reduced between age‐matched Sham and AC groups. ^***^ *P* < 0.001. Sham *n* = 22 and 150AC *n* = 46 for NKAα1, Sham *n* = 49 and 150AC *n* = 30 for NKAα2 from two hearts per group. Mean (SD).

Figure 8 *A* shows typical staining patterns obtained from immunolabeling of α1 (Fig. 8 *Aa*) and α2 (Fig. 8 *Ab*) Na^+^,K^+^‐ATPase isoforms in cells isolated from 150‐day sham‐operated controls and 150‐day AC hearts. To assess the changes in α1 and α2 isoform staining we created two regions of interest. The staining intensities within these areas were calculated and grouped to yield pooled measurements from at least 10 cells from two hearts of each group. These data are shown in Fig. 8 *B*. The overall staining was greater in sham cells compared with the HF cells.

**Figure 9:**
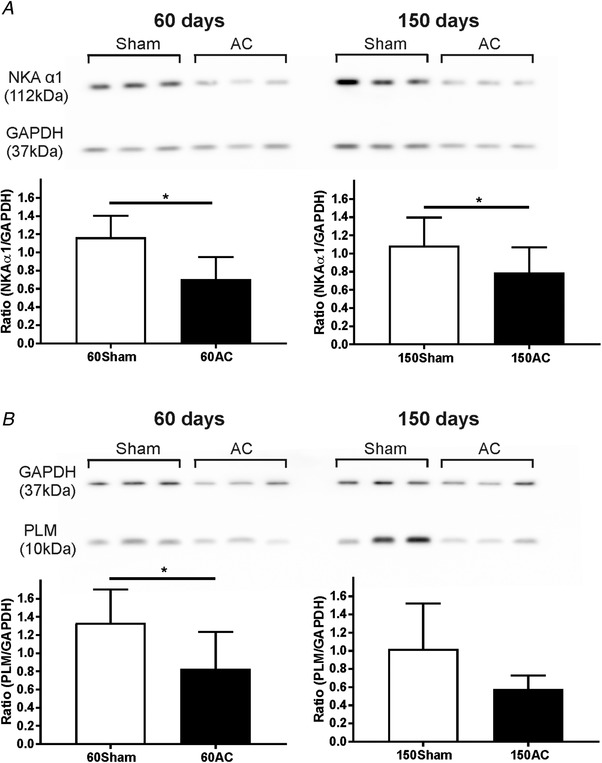
**Western blot analyses of Na^+^, K^+^ ATPase α1 and phospholemman between animal groups** *A*, western blot of Na^+^,K^+^‐ATPase (NKA) α1 expression in Sham and AC groups at 60 and 150 days post‐operatively. The Na^+^,K^+^‐ATPase α1 relative expression was significantly reduced in 60 and 150 days compared to their age‐matched Sham groups. *N* = 5 for 60Sham and *N* = 4 for 60AC; *N* = 6 for 150Sham, *N* = 7 for 150AC. Mean (SD). Student's *t* test one‐tailed, ^*^ *P* < 0.05, ^**^ *P* < 0.01. *B*, western blot of expression of phospholemman (PLM), a protein which regulates the activity of Na^+^,K^+^‐ATPase. The relative expression of PLM significantly decreases between 60‐day sham and 60‐day AC, but was unchanged between the 150‐day AC group compared to age‐matched Sham. Mean (SD). Student's *t* test one‐tailed, ^*^ *P* < 0.05. *N* = 5 for 60Sham and *N* = 7 for 60AC; *N* = 4 for 150Sham, *N* = 4 for 150AC.

Both the reduction in function and in immunolabeling of the Na^+^,K^+^‐ATPase are mirrored by the decline in protein levels after 60 and 150 days as assessed by western blotting. This result is shown in Fig. 9 *A*. GAPDH was used as the loading control and blot densities were normalized to the level of this protein. The 60‐day and 150‐day AC groups had significant decreases in the mean protein levels of the α1 isoform compared with the age‐matched sham groups. The levels of α2 isoform were unchanged.

The Na^+^,K^+^‐ATPase is regulated by phospholemman (PLM) and so we assessed the levels of this protein (also normalized to GAPDH) in the same samples (Fig. 9 *B*). There was a decline in expression between Sham and AC at 60 days; however, there was no difference in PLM protein expression between 150‐day sham and 150‐day AC groups.

### Physiological changes following the decline in Na^+^,K^+^‐ATPase function

Our Na^+^,K^+^‐ATPase functional data suggest there is about a 50% reduction in pump current in the compensated stage of heart failure. We investigated if this reduction could act as a positive inotropic mechanism to preserve contractile function early in the progression of disease and be sufficient to explain the enhanced SR content and larger Ca^2+^ transients measured at that stage.

**Figure 10:**
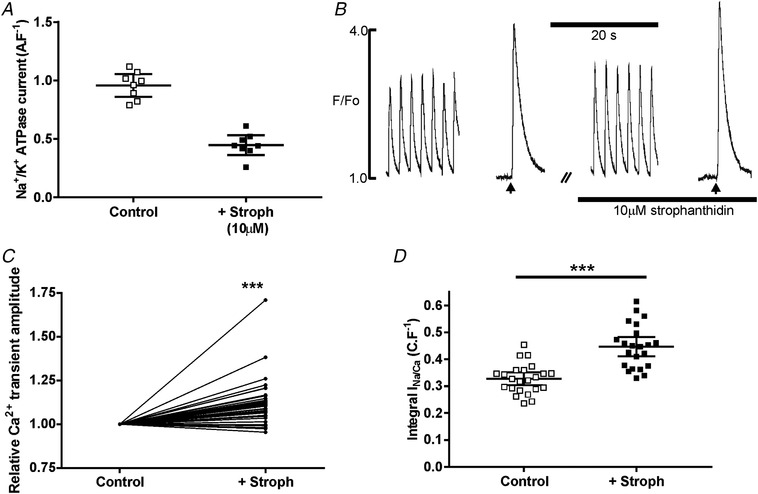
**The effects of an approximately 50% inhibition of Na^+^, K^+^‐ATPase function with 10 μm strophanthidin on the Ca^2+^ transient and SR Ca^2+^ content of myocytes isolated from sham‐operated hearts** *A*, application of 10 μm strophanthidin produced a 47 ± 7% reduction of Na^+^,K^+^‐ATPase function (assessed using 8 cells isolated from 3 hearts); scatter plot shows mean (95% CI). *B*, typical effect of 10 μm strophanthidin on the electrically stimulated and caffeine‐evoked Ca^2+^ transients (caffeine was applied at the arrows). *C*, pooled data from these experiments; ^***^ *P* < 0.001; *N* = 38/4. *D*, pooled data from voltage clamp experiments measuring Na^+^/Ca^2+^ exchange current on application of caffeine during protocols similar to that illustrated in *B*. Student's *t* test, ^***^ *P* < 0.001, *n*/*N* = 21/4. Scatter plots show mean (95% CI).

Following a series of dose–response experiments (Fig. 10 *A*), we found that application of 10 μm strophanthidin produced a 47 ± 7% reduction of Na^+^,K^+^‐ATPase current. This reduction in current increased the Ca^2+^ transient amplitude by 12% (Fig. 10 *C*) and the integral of caffeine‐induced inward Na^+^/Ca^2+^ exchange current by 37% (Fig. 10 *D*). These results suggest that inhibition of the Na^+^,K^+^‐ATPase current by a similar amount to that at the compensated hypertrophy stage can account for the increase in SR load and the maintenance of the Ca^2+^ transient. In the setting of developing HF, this could maintain cardiac function that would otherwise be in decline.

**Figure 11:**
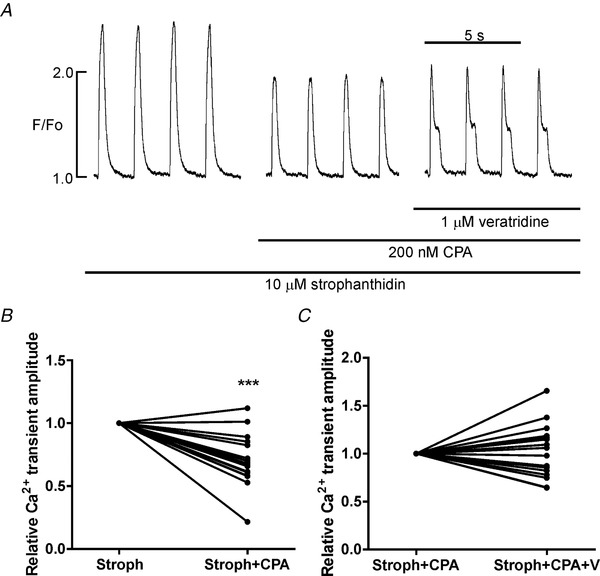
**Increasing Na^+^ influx following partial inhibition of the Na^+^, K^+^‐ATPase and SERCA function does not increase the amplitude of the Ca^2+^ transient** *A*, effects on the Ca^2+^ transient, recorded in myocytes isolated from healthy hearts, of about 50% inhibition of SERCA function with 200 nm cyclopiazonic acid (CPA) when the Na^+^,K^+^‐ATPase has also been inhibited (10 μm strophanthidin, Stroph). The time between the first two traces is 3 min. *B*, pooled data from these experiments indicating the Ca^2+^ transient amplitude decreases by 30%. Student's *t* test, ^***^ *P* < 0.001, *n*/*N* = 18/2. In the experiment shown in *A*, further addition of 1 μm veratridine (V) to increase Na^+^ influx results in aberrant Ca^2+^ release and pooled data from these experiments (*C*) shows no increase in Ca^2+^ transient amplitude, *n*/*N* = 17/2. The time between the second two traces is 2 min.

We also investigated if this inotropy could be altered by partial inhibition of SERCA in healthy cells so reproducing the gradual failure of SR Ca^2+^ uptake that occurs in the progression to failure. When Na^+^,K^+^‐ATPase was reduced by 50%, an inhibition of SERCA function also by about 50% using 200 nm cyclopiazonic acid (Schwinger *et al*. 1997) reduced Ca^2+^ transients by 30% (Fig. 11 *A* and *B*). Increasing Na^+^ influx using 1 μm veratridine in this condition did not augment Ca^2+^ transients and was pro‐arrhythmic (Fig. 11 *A* and *C*).

### Action potential duration

**Figure 12:**
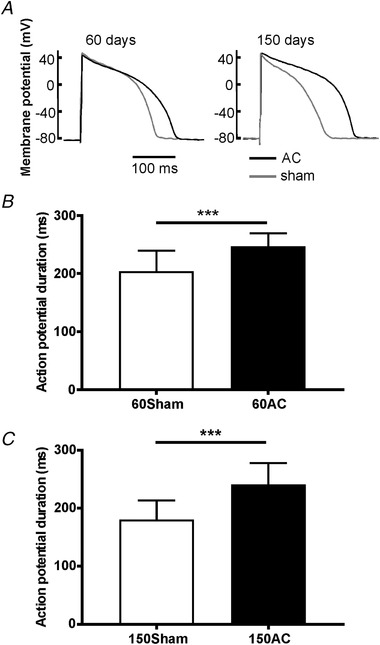
**Mean action potential durations at 90% repolarization measured in ventricular myocytes isolated from hearts 60 and 150 days after AC** *A*, sample traces. *B* and *C*, pooled data for 60 days (*B*) and for 150 days (*C*). Means (SD); *n* = 26, 11, 31 and 11 for 60Sham, 60AC, 150Sham and 150AC, respectively, from 4–8 animals. Student's *t* test, ^***^ *P* < 0.001.

A consistent finding in cardiac hypertrophy and heart failure in many studies is prolongation of the action potential duration (APD) (Beuckelmann *et al*. 1993; Kaab *et al*. 1996; Milnes & MacLeod, 2001; Pogwizd *et al*. 2001) likely due to changes in the expression and function of ion channels and transporters. We found prolongation of the action potential occurred early in the progression of disease and APD remained prolonged in the later stages (Fig. 12).

### Late Na^+^ current

**Figure 13:**
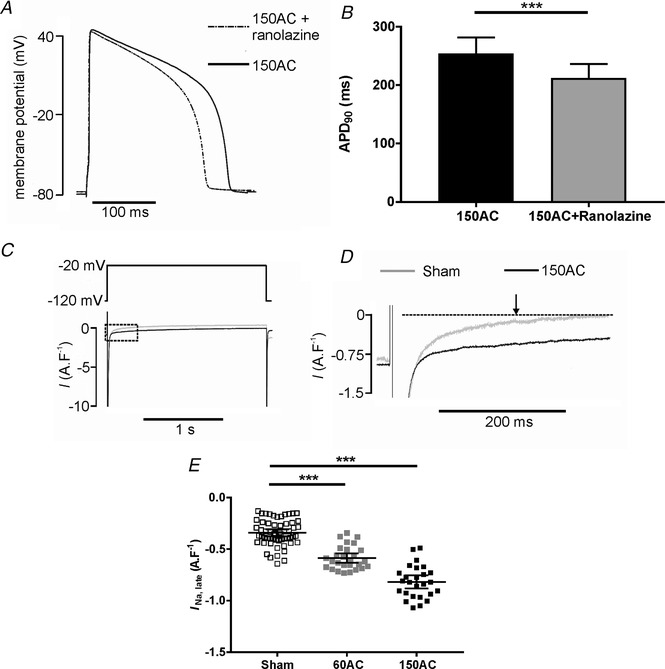
**Changes in INa, late during progression towards HF** *A*, APD was shortened in the presence of 10 μm ranolazine in a cardiac myocyte from the 150AC group of animals. *B*, mean data showing APD_90_ in cardiac myocytes from 150AC group was significantly shortened in the presence of 10 μm ranolazine (150AC, *n*/*N* = 23/6; mean (SD), Student's *t* test, ^**^ *P* < 0.001). *C* and *D*, typical *I*_Na,late_ traces in 150Sham and 150AC cells. *E*, scatter plot of pooled data with mean (95% CI) for each group. There were no changes in *I*_Na,late_ in the age‐matched sham myocytes so these sham data were combined. ^***^ *P* <  0.001; *n* = 58, 27 and 26 from 4–8 hearts for Sham, 60AC and 150AC respectively. One‐way ANOVA with a Sidak *post hoc* test.

A second component of Na^+^ current that persists during the action potential is known as the late Na^+^ current (*I*_Na,late_). Although very small in comparison to the main Na^+^ current that produces the AP upstroke, its persistence ensures it contributes to the plateau phase potential, morphology and duration and to Na^+^ influx. There is evidence that the density of the current increases and its inactivation kinetics slow in heart failure (Maltsev *et al*. 2007). We tested the effect of ranolazine, a compound shown to inhibit *I*_Na,late_ (Antzelevitch *et al*. 2004), on APD and found that 10 μm shortened APD by about 16% in myocytes isolated from 150AC hearts (Fig. 13 *A* and *B*).

We measured ranolazine‐sensitive *I*_Na,late_ 215 ms from the start of 2 s depolarization steps from a holding potential of –120 mV to – 20 mV (arrow in Fig. 13 *D*). *I*_Na,late_ was increased in the compensated stage (60 days) and further increased during disease progression so, when the failing stage was reached, it was about 240% larger than control.

## Discussion

### Whole heart observations

In this study we follow the development of cardiac cellular Ca^2+^ pathology from the initial stages of cardiac overload and the formation of compensated hypertrophy, until signs of heart failure are apparent. We do so in an animal whose cardiac cell physiology closely resembles that of the human. It presents a unique insight into the progressive changes that take place that, when studied in isolation, may give rise to areas of confusion and contradiction. We considered a variety of indices that, in our view, are important markers of whole heart function and intracellular Ca^2+^ and Na^+^ regulation, to build a picture of the progression of cellular Ca^2+^ pathology. Our results suggest a continuum of change with the earliest modifications being a reduction in Na^+^,K^+^‐ATPase current and an increase in late Na^+^ current.

The guinea‐pig animal model of pressure overload due to aortic constriction (AC) has already been used in a number of studies mainly at the point where cardiac hypertrophy is well developed – between 40 and 60 days post‐operatively (Siri *et al*. 1991; Jelicks & Siri, 1995; Kingsbury *et al*. 2000; Gray *et al*. 2001; McGoldrick *et al*. 2007). Heart‐to‐body weight ratio, the commonly used index of cardiac hypertrophy, indicates that significant hypertrophy (an increase of around 50%) has developed by this time (similar in extent to that found by Kingsbury *et al*. (2000) and Gray *et al*. (2001) using the same model). Indices of left ventricular systolic function and dilatation (FS and LVIDs) do not change and the cellular Ca^2+^ transient increases, emphasizing the establishment of compensation. At 150 days post‐AC these indices demonstrate a decline in whole heart function. While the HW:BW ratio remains greater, FS decreases to 57% of control while LVIDs increases about 2.8‐fold. In addition, there is an increase in LVIDd suggesting volume retention in the left ventricle, which is likely to produce pulmonary congestion, presumably the cause of the increased lung weight‐to‐body weight ratio measured at this stage.

The profound decline in whole heart function parallels a decrease in the size of cellular Ca^2+^ transients (to 60% of control), their prolongation, and a decrease in SR Ca^2+^ content, three hallmarks of the cellular phenotype of HF in animal and human studies (Gwathmey & Morgan, 1985; Gwathmey *et al*. 1987; Beuckelmann *et al*. 1992; del Monte *et al*. 1995; Hasenfuss, 1998).

### Compensative inotropy and Na^+^,K^+^‐ATPase function

We found that the preserved contractile function early in the progression of disease occurred concurrently with enhanced SR content, larger Ca^2+^ transients, a decrease in the amount and function of Na^+^,K^+^‐ATPase and an increase in late Na^+^ current. Earlier work using this animal model found that at this compensated hypertrophy (60‐day) stage, bulk cytoplasmic [Na^+^] had increased by 4–5 mm (Gray *et al*. 2001) in keeping with other studies of hypertrophy (Jelicks & Siri, 1995) and heart failure (Despa *et al*. 2002; Pieske *et al*. 2002; Baartscheer *et al*. 2003; Schillinger *et al*. 2006; Liu *et al*. 2014).

The main causes of the cytosolic Na^+^ accumulation remain uncertain. We have not measured all the individual inward Na^+^ fluxes that may contribute but focused on one that has been demonstrated previously to increase in HF – *I*_Na,late_ (Valdivia *et al*. 2005; Maltsev & Undrovinas, 2008). We found that when the compensated stage has been reached, *I*_Na,late_ has increased by about 70% and increases more as the disease progresses. The modelling work by Cardona *et al*. (2016) suggests that changes in [Na^+^]_i_ due to increases in *I*_Na,late_ of the magnitudes we observe are very small (about 0.1 mm). We cannot rule out the contribution of other transport processes (Despa *et al*. 2002) but, given the simulation work of Cardona and colleagues and our observation that the same degree of inotropy we observe in the compensated state is reproduced in the healthy cell by inhibiting the Na^+^,K^+^‐ATPase by about 50%, we conclude that a major cause of Na^+^ loading is a reduction in Na^+^,K^+^‐ATPase function.

The increase in [Na^+^]_i_ found in this model at the compensated hypertrophy stage of the disease process (from 7 to 12 mm, Gray *et al*. 2001) will alter the balance of Ca^2+^ flux generated by the Na^+^/Ca^2+^ exchange. Coupled with the longer action potential duration (Fig. 12), the exchange will become biased towards reduced forward mode and increased reverse mode which could mediate the observed increases in Ca^2+^ transients and SR Ca^2+^ contents (Figs 2 and 4). The increase in SR Ca^2+^ content will tend to promote increased Ca^2+^ release that will limit early contractile impairment. We suggest that these changes in Ca^2+^ flux, driven by the decline in Na^+^,K^+^‐ATPase function, could be important for establishing a compensated state. As the hearts progress towards failure, this compensation declines, because the increase in SR Ca^2+^ content cannot be sustained as SERCA function declines (see Fig. 3 *C*) and SR Ca^2+^ leak increases (see Fig. 5 *F*).

To support these suggestions, we have shown that simply inhibiting the Na^+^,K^+^‐ATPase function in normal cardiac myocytes by a similar amount as measured in the 60‐day AC heart cells accomplishes a similar enhancement of Ca^2+^ transients and increase in SR Ca^2+^ content (see Fig. 10). Indeed, Na^+^,K^+^‐ATPase inhibitors (glycosides including digoxin) were one of the earliest treatments for the failing heart following the work of Withering, who described the advantages of administering extracts of the foxglove to patients with HF (Hauptman & Kelly, 1999). The result of a reduction in SERCA function following inhibition of Na^+^,K^+^‐ATPase function in otherwise healthy cardiac myocytes results in a decline in Ca^2+^ transients by 30% (see Fig. 11 *A* and *B*). It is notable that increasing Na^+^ influx in this condition did not augment Ca^2+^ transients but instead was pro‐arrhythmic (see Fig. 11 *A* and *C*).

There have been very few studies that have measured Na^+^,K^+^‐ATPase function in hypertrophy and HF and this is the only one to do so in both pathologies. The consensus finding is that function declines (Semb *et al*. 1998; Gray *et al*. 2001; Swift *et al*. 2008; Louch *et al*. 2010), though this may be because of a reduced affinity for Na^+^, rather than a decrease in maximum pump current (Verdonck *et al*. 2003 *a*). In this study we observed that the decline in function happens early in the hypertrophic process and remains low as HF develops.

The currents due to the α1 and the α2 Na^+^,K^+^‐ATPase isoforms both decline by similar percentages. Verdonck *et al*. (2003 *a*) also found dihydro‐ouabain sensitivity was not altered in cardiac myocytes isolated from a canine chronic atrioventricular block model of HF. Our data suggest that the α2‐isoform in guinea pig ventricular myocytes accounts for a larger proportion of the total current (around 43%) than in Wistar rat heart cells used in the study of Swift *et al*. (2007). The immunofluorescence experiments and western blots reinforce the findings of reduced Na^+^,K^+^‐ATPase current. There is less fluorescence staining in the centre of the cells presumably in areas associated with t‐tubules, but both α1 and α2 isoforms are still expressed. These general features are similar to the staining patterns for guinea pig ventricular myocytes found by Silverman *et al*. (2005). Our immunocytochemical images and western blots allow us to conclude that the amounts of α1 and α2 isoforms decrease in failing myocytes compared with those isolated from sham operated hearts. Qualitative changes in isoform ratio are not apparent, and therefore we cannot infer that downstream alterations to Ca^2+^ regulation are more dependent on one isoform.

It is now recognized that in the heart the Na^+^,K^+^‐ATPase comprises a complex of α1 or α2 (or in human, α3) with β1 and FXYD‐1 or γ subunits. The β1 subunit supports protein trafficking to the membrane and FXYD‐1, usually known as phospholemman (PLM), modulates the Na^+^,K^+^‐ATPase function depending on the amount of its phosphorylation. Unphosphorylated PLM inhibits the cardiac Na^+^,K^+^‐ATPase while phosphorylated PLM stimulates it (see Pavlovic *et al*. (2013) for review). El‐Armouche *et al*. (2011) suggest that PLM dephosphorylation (and consequent Na^+^,K^+^‐ATPase deactivation) occurs in hypertrophy mainly through changes to protein phosphatase‐1 activity. We found total PLM expression variable and this might be an explanation for our not being able to observe uniform changes in total PLM during progression towards HF. This is consistent with El‐Armouche *et al*. (2011) who found reduced PLM phosphorylation at Ser68 but no change in total PLM in HF and contradictory to the findings of Bossuyt *et al*. (2005) who reported a reduction in total PLM (and α1 subunit expression) in a rabbit heart failure model and an increase in PLM phosphorylated at Ser68.

Although the increase in *I*_Na,late_ may not contribute greatly to the increase in bulk cytosolic [Na^+^], our work shows that this Na^+^ influx is associated with AP prolongation. This may be more important in influencing trans‐sarcolemmal Ca^2+^ fluxes and SR Ca^2+^ content compared with its role as a source of Na^+^ influx because APD determines both fluxes and content (Terracciano *et al*. 1997).

### SR function in early and late disease stages

The increase in SR Ca^2+^ content will depend on adequate SERCA and ryanodine receptor function. The Ca^2+^ transients decay at similar rates at the compensated stage and our data suggest that the relative contributions of SERCA, Na^+^/Ca^2+^ exchange and other slower transporters to Ca^2+^ removal remain unchanged allowing SR Ca^2+^ content to be increased.

By 150 days, uptake by SERCA was slowed and the relative contributions of SERCA and the Na^+^/Ca^2+^ exchange to Ca^2+^ removal were markedly changed. These changes occurred in parallel with modifications to the SR Ca^2+^ release channels that resulted in more Ca^2+^ leak (Marks, 2000; Marx *et al*. 2000). Together these alterations could be predicted to result in the observed reduction in SR Ca^2+^ content and smaller and slower Ca^2+^ transients.

### Limitations

The work demonstrates that the decline in Na^+^,K^+^‐ATPase function early in the hypertrophic process shapes the compensated state by helping to load the SR with Ca^2+^ until the disease state progresses to the point when SERCA function declines and the SR load cannot be sustained. For the initial compensation to occur, cytosolic [Na^+^] needs to increase and one limitation of the study is that we can only infer that this happens albeit from a position of relative strength. Cytosolic [Na^+^] concentrations have been measured by others using exactly this model of hypertrophy and HF (Gray *et al*. 2001) and in a similar model of aortic constriction in the guinea pig (but using a different method whereby HF develops more quickly (Liu *et al*. 2014)). Both these papers indicate an increase in cytosolic [Na^+^] of 5–10 mm in the respective disease states in line with many other measurements on a variety of species (see Verdonck *et al*. (2003 *b*) for review). Another limitation is that changes in bulk cytosolic [Na^+^] may not reflect alterations in subsarcolemmal [Na^+^] regulation. It is not inconceivable that there will be differences in co‐localization of ion transporters in disease states that will create microdomains with different functional characteristics.

The increase in cytosolic [Na^+^] biases the bidirectional function of the Na^+^/Ca^2+^ exchange, increasing reverse mode and reducing forward mode operation during the cardiac cycle, particularly when APD is prolonged. During the compensated hypertrophy stage, the Ca^2+^ transients recorded from myocytes isolated from 60‐day AC hearts were larger than those isolated from sham‐operated control cells and we suggest that this effect is due to increased Ca^2+^ influx by the Na^+^/Ca^2+^ exchange that contributes to the transient and also to the observed increase in SR Ca^2+^ content. This occurs with no change to the rate constant of exchange‐mediated Ca^2+^ removal. Whilst the rate constants for Ca^2+^ removal allow a comparison of the contributions of the various Ca^2+^ regulatory systems to relaxation, they need to be viewed with some caution. It is presumed that the rate constants should be independent of the peak value of [Ca^2+^]_i_, i.e. the transport rates are linear functions of free [Ca^2+^]_i_. However, Bers & Berlin (1995) demonstrated that, because there is a non‐linear relationship between free [Ca^2+^]_i_ and fluxes through the regulatory proteins, such rate constants are dependent on the range of free [Ca^2+^]_i_ over which they are measured. The Ca^2+^ transients recorded from myocytes isolated from compensated hearts are larger so the rate constants could be faster than expected simply on thermodynamic grounds.

The non‐linear relationship between free [Ca^2+^]_i_ and fluxes therefore imposes limitations on some of the interpretations of our data. Calibration of the cytosolic Ca^2+^ changes using a ratio‐able dye would have allowed intracellular Ca^2+^ buffering calculations (Trafford *et al*. 1999), subsequent quantitative transformation of free [Ca^2+^]_i_ to total [Ca^2+^] and so more complete assessment of the Ca^2+^ fluxes by SERCA, Na^+^/Ca^2+^ exchange and the slow systems (sarcolemmal Ca^2+^‐ATPase and mitochondrial Ca^2+^ uniporter) (Bers *et al*. 1996). Calibration would also have been beneficial in assessing the extent of diastolic Ca^2+^ increases. Such increases are intrinsically positively inotropic but the contribution that such changes may make to the maintenance of force and therefore fractional shortening in the whole heart in the compensation state is uncertain.

There is a large amount of evidence that the calcineurin–nuclear factor of activated T cells signalling network coupled with other pathways such as those involving mitogen‐activated protein kinase are central to activation of the hypertrophic response. It is thought that increased diastolic [Ca^2+^] (or low‐amplitude oscillations of Ca^2+^) provide an activation signal for calcineurin (Molkentin, 2004). However, the signalling systems that may trigger changes in the distinct phases of compensation and decompensation are uncertain. Our focus has been on the intracellular Na^+^ control of the sarcolemmal Na^+^/Ca^2+^ exchange but [Na^+^]_i_ also determines mitochondrial [Ca^2+^] ([Ca^2+^]_m_). Mitochondrial Ca^2+^ homeostasis is a balance of Ca^2+^ influx through the mitochondrial uniporter and efflux through the mitochondrial Na^+^/Ca^2+^ exchange. An increase in cytoplasmic [Na^+^] causes a decrease in [Ca^2+^]_m_ and reduces oxidative phosphorylation so slowing mitochondrial ATP production (Iwai *et al*. 2002; Maack *et al*. 2006). There is growing evidence that HF involves impaired cellular energy production (Brown *et al*. 2017) and the onset of this impairment may mark a transition from compensation to a decompensated state.

## Conclusions

Our aims with the work detailed here were to follow the changes in cellular Ca^2+^ and Na^+^ regulation from the initial stages of cardiac overload and the formation of compensated hypertrophy, until signs of heart failure are apparent in an animal whose cardiac cell physiology closely resembles that of the human.

We provide strong evidence to suggest that there is a decline in Na^+^,K^+^‐ATPase function that happens early in the hypertrophic process. This may be important in shaping the compensated state by helping to load the SR with Ca^2+^ until later in the progression of the disease state when SERCA function declines, the SR becomes leaky and SR Ca^2+^ content declines.

## Additional information

### Competing interests

No author involved in this work has any conflict of interest that they need to declare.

### Author contributions

Authors have made the following contributions to the work: conception and design H.‐Y.K., H.‐Y.Y., A.F., T.C., J.F., K.M.; acquisition, analysis and interpretation of data H.‐Y.K., H.‐Y.Y., A.F., T.C., H.S., A.A.‐L., J.F., K.M.; drafting the work or revising it critically for important intellectual content H.‐Y.K., H.‐Y.Y., A.F., A.A.‐L., J.F., K.M. All authors have read and approved the final version of this manuscript and agree to be accountable for all aspects of the work in ensuring that questions related to the accuracy or integrity of any part of the work are appropriately investigated and resolved. All persons designated as authors qualify for authorship, and all those who qualify for authorship are listed.

### Funding

This work was supported by the British Heart Foundation [Project Grant Number: PG/032/27241] to K.T.M. H.‐Y.K. and H.‐Y.Y. received funding from Tri‐Service General Hospital, National Defense Medical Center, Taipei, Taiwan (ROC). We also acknowledge use of the Imperial College Facility for Imaging by Light Microscopy (FILM).
